# Supplementary material for: Remapping the spatial distribution of neutralizing sites and their immunodominance on the capsid of different topotypes of FMDV serotype O by site-directed competitive ELISA for detection of neutralizing antibodies
Source: Microbiol Spectr. 2025 May 15;13(6):e03344-24. doi: 10.1128/spectrum.03344-24 (PMC12131818; doi:10.1128/spectrum.03344-24)
Supplement: Supplemental figures and tables — Fig. S1 to S3 and Tables S1 to S8. [file spectrum.03344-24-s0001.docx]

**Figures S1 to S3**


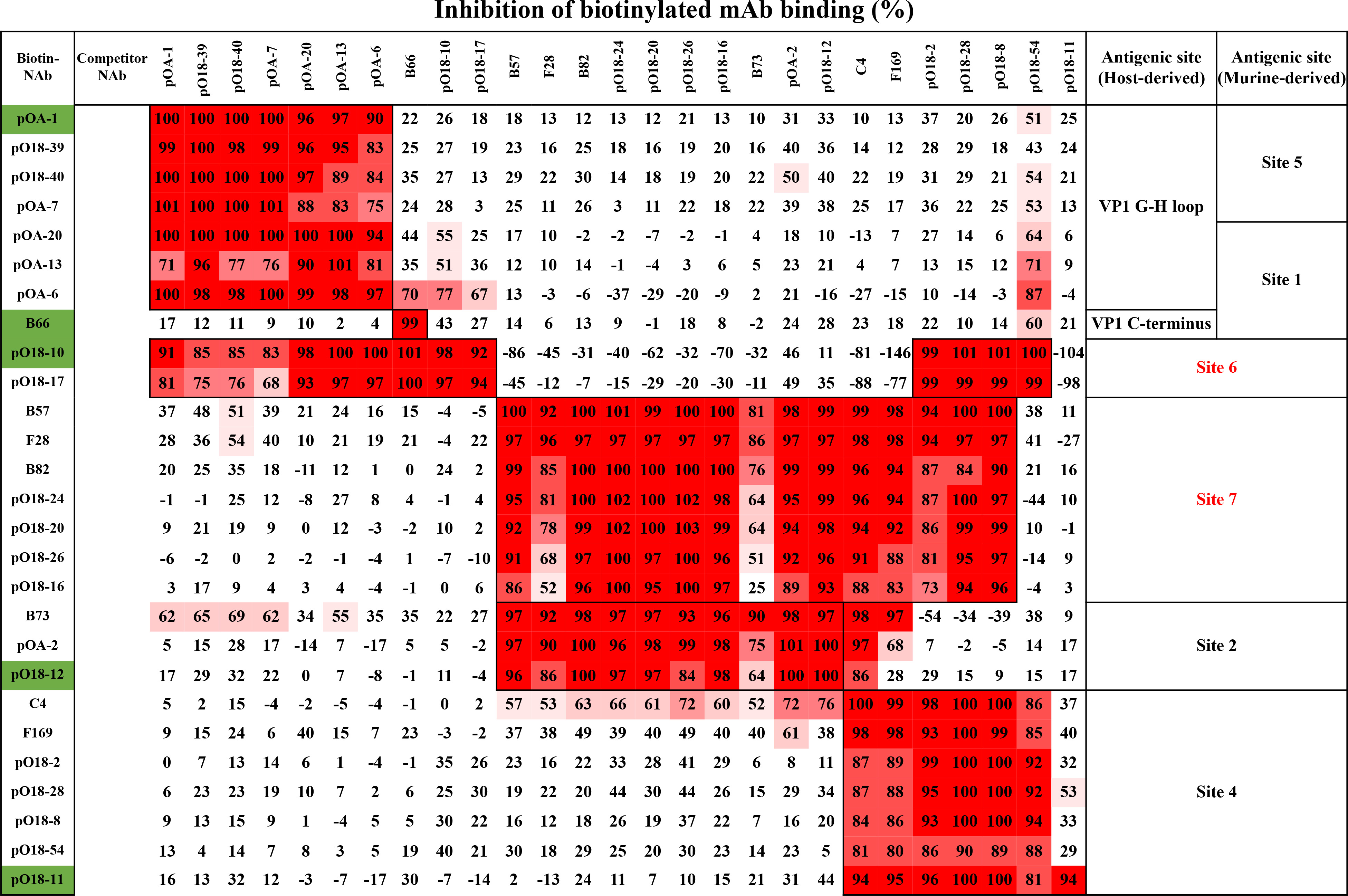


**Fig. S1.** **E32 captures the O/Tibet/99 antigen for pairwise cELISA and categorizes 27 host-derived antibodies into six classes based on their competitive relationships.** A gradually enhanced red color was used to visualize an increasing percentage inhibition rate of Biotin-NAbs binding.


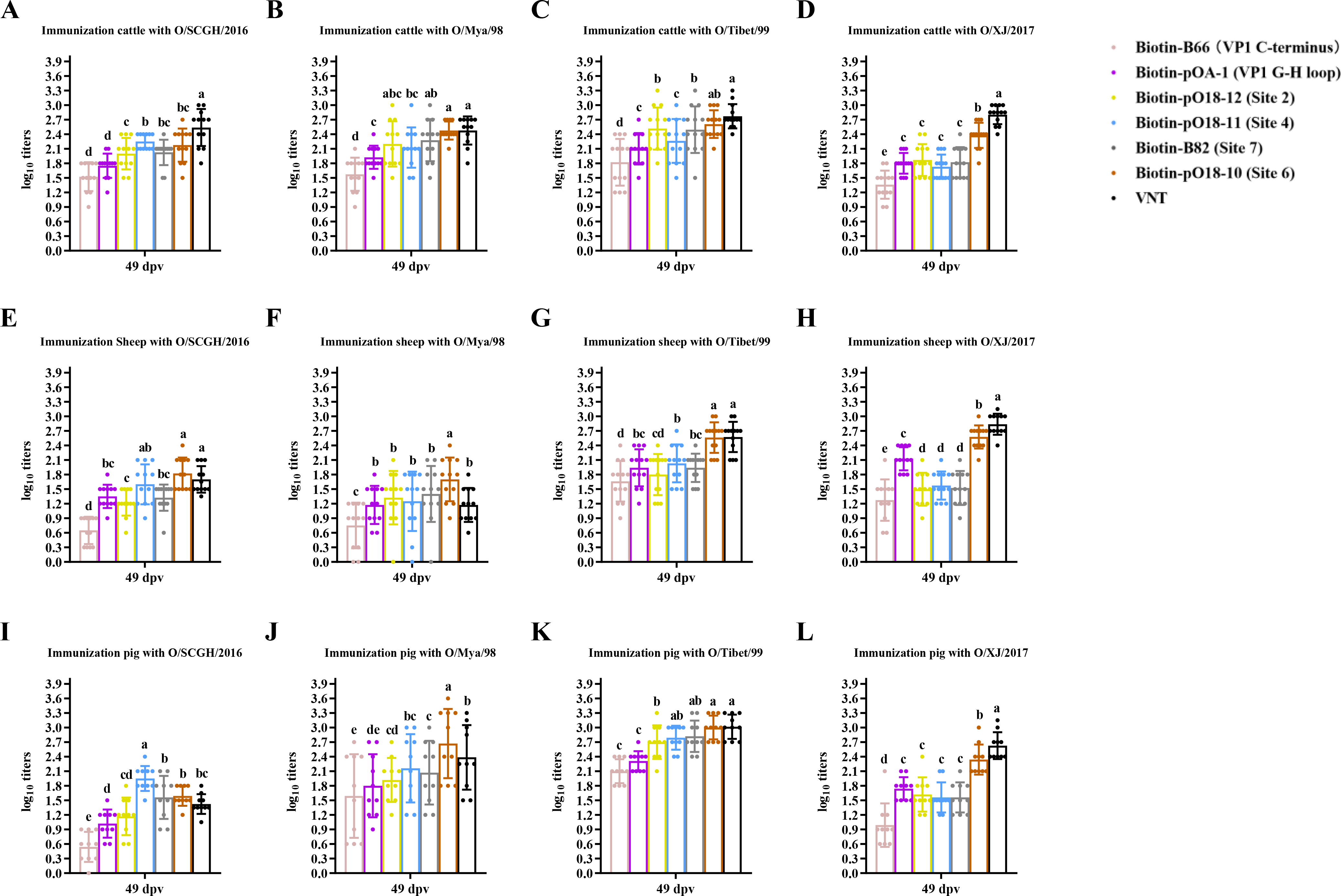


**Fig. S2.** **Comparison of antibody abundance against various neutralizing sites following immunization of three hosts with four lineages of FMDV serotype O vaccines.** Using the E32-captured O/Tibet/99 antigen, we compared antibody abundance to various neutralizing sites after the immunization of cattle (A-D), sheep (E-H), and pigs (I-L) with four lineages of FMDV serotype O vaccines: O/SCGH/2016, O/Mya/98, O/Tibet/99, and O/XJ/2017 at 49 dpv after booster immunization. Antibody abundance was assessed using a site-directed cELISA targeting six sites: Biotin-B66 (VP1 C-terminus), Biotin-pOA-1 (VP1 G-H loop), Biotin-pO18-12 (site 2), Biotin-pO18-11 (site 4), Biotin-B82 (site 7), and Biotin-pO18-10 (site 6). Total NAb titers to O/Tibet/99 virus at 49 dpv were determined through a VNT test. Error bars represent 95% confidence intervals. A one-way ANOVA (Tukey's multiple comparisons test) was conducted to assess the statistical significance of differences for antibody titers to different sites within the same lineage, with distinct lowercase letters indicating significant differences for each data point in the 49 dpv sera (*P* < 0.05).


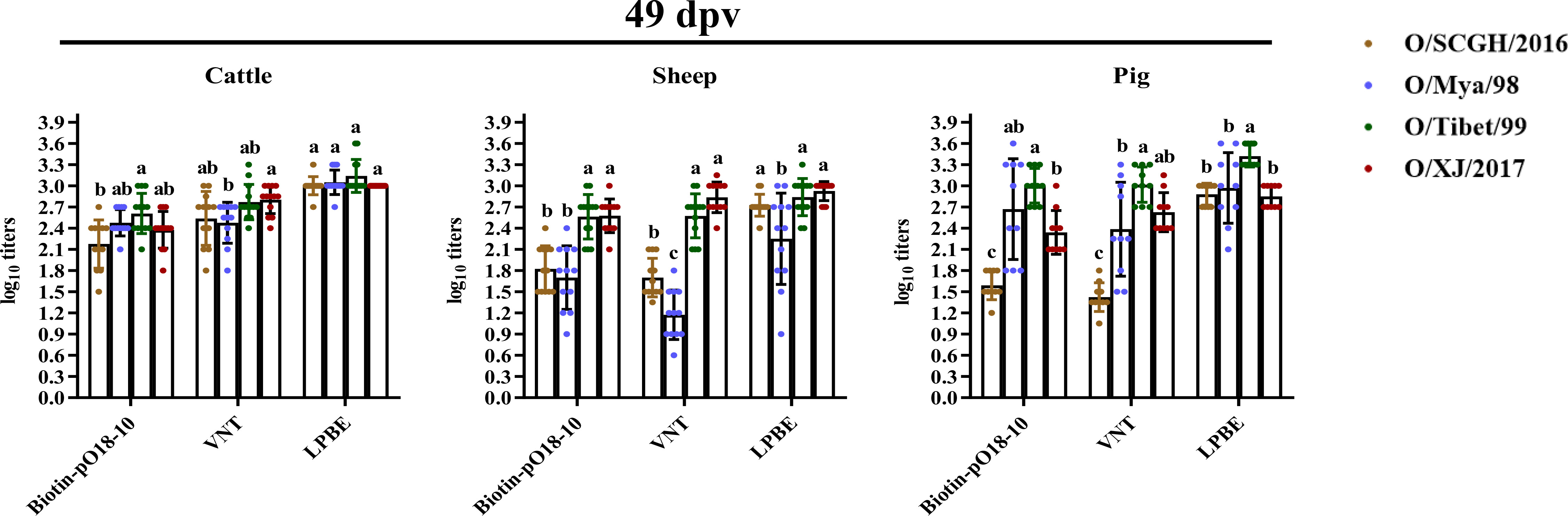


**Fig. S3.** **Immunogenicity analysis of four lineages of FMDV serotype O antigens in different hosts.** Serum samples were collected from cattle, sheep, and pigs at 49 dpv after primary immunization to analyze the immunogenicity of four lineages of FMDV serotype O vaccines: O/Mya/98 (SEA topotype), O/SCGH/2016 (Cathay topotype), O/Tibet/99, and O/XJ/2017 (ME-SA topotype). A one-way ANOVA (Tukey's multiple comparisons test) was performed to assess differences in cELISA results for Biotin-pO18-10 (E32 captured O/Tibet/99), VNT results (O/Tibet/99), and LPBE results at 49 dpv. Error bars represent 95% confidence intervals. In each figure, different letters indicate significant differences between the strains during pairwise comparisons of the four topotype strains (*P* < 0.05).

**Table S1 to S8**

**Table S1. Critical residues of antigenic sites recognized by 35 bovine or porcine originated monoclonal neutralizing antibodies used in this study.**

| Classical antigenic site | Host | NAb | Critical residues |
| --- | --- | --- | --- |
| Site 1 | Pig | pOA-6 | VP1-133, 137, 138, 148 |
|  | Pig | pOA-13 | VP1-133, 138, 148 |
|  | Pig | pOA-20 | VP1-148 |
|  | Pig | pO18-8 | VP1-108, 204 |
|  | Pig | pO18-10 | VP1-99, 143, 158, 204; VP3-173, 174 |
|  | Pig | pO18-17 | VP1-99, 204 |
|  | Pig | pO18-52 | VP1-204; VP3-173 |
|  | Pig | pO18-53 | VP1-204; VP3-173 |
|  | Pig | pO18-57 | VP1-158, 207 |
|  | Cattle | B66 | VP1-207, 209, 211 |
| Site 2 | Cattle | B57 | VP2-71, 72 |
|  | Cattle | B73 | VP2-72 |
|  | Cattle | B77 | VP2-190 |
|  | Cattle | B82 | VP2-71, 72 |
|  | Cattle | F28 | VP2-71, 72 |
|  | Pig | pOA-2 | VP2-68 |
|  | Pig | pO18-12 | VP2-68, 77, 196 |
|  | Pig | pO18-16 | VP2-71, 188, 195; VP3-196, 197, 209 |
|  | Pig | pO18-20 | VP2-71, 72, 188, 190, 195; VP3-197 |
|  | Pig | pO18-24 | VP2-188, 190, 195 |
|  | Pig | pO18-26 | VP2-71, 195 |
| Site 3 | Cattle | A19 | VP1-58 |
|  | Cattle | B74 | VP1-43, 58 |
|  | Cattle | C5 | VP1-58 |
|  | Cattle | E18 | VP1-43 |
| Site 4 | Cattle | C4 | VP3-65, 68, 69, 131, 134, 195, 196 |
|  | Cattle | F169 | VP3-68 |
|  | Pig | pO18-2 | VP3-70, 76, 131, 134; VP1-199 |
|  | Pig | pO18-11 | VP3-131 |
|  | Pig | pO18-28 | VP3-58, 61, 70, 76; VP1-108 |
|  | Pig | pO18-54 | VP3-174, 209; VP1-194, 204 |
| Site 5 | Pig | pOA-1 | VP1-133, 138, 149 |
|  | Pig | pOA-7 | VP1-149 |
|  | Pig | pO18-39 | VP1-143, 149 |
|  | Pig | pO18-40 | VP1-149 |

**Table S2. Critical residues of conventional antigenic sites of FMDV serotype O identified with mouse derive monoclonal neutralizing antibodies.**

| Classical antigenic site | Host | NAb | Critical residues | References |
| --- | --- | --- | --- | --- |
| Site 1 | Mouse | D9 | VP1-144, 148, 154, 208 | Haresnape et al.,1983; |
| Site 2 | Mouse | C6 | VP2-70-73, 75, 77, 131 | Mccullough et al., 1987; |
| Site 3 | Mouse | C8 | VP1-43, 44, 45 | Kitson et al., 1990; |
| Site 4 | Mouse | 14EH9 | VP3-56, 58 | Crowther et al., 1993; |
| Site 5 | Mouse | C3 | VP1-149 | Barnett et al., 1998. |

**Table S3. Optimized conditions for developing the sites-directed cELISAs.**

| Steps of site-directed NAbs cELISA | Optimized dilutions and reaction conditions |
| --- | --- |
| Coating condition | 0.5 μg/ml in E32 |
|  | 4℃, 14 h |
| Capturing condition | 1 μg/ml in 146S antigen (O/Mya/98 or O/Tibet/99) |
|  | RT, 2 h |
| Blocking condition | 5% sucrose and 1% BSA in PBS |
|  | 37℃, 1 h |
| 35 strains Biotin-NAbs | 2-fold dilutions starting at 5 μg/mL, respectively |
|  | 37℃, 1 h |
| The HRP-conjugated streptavidin antibodies | 1:30,000 |
|  | 37℃, 15 min |
| Tetramethylbenzidine (TMB) chromogenic substrate | 100 μl |
|  | 37℃, 15 min |

**Table S4. Concentrations of 27 Biotin-NAbs suitable for cELISA.**

| NAb | Concentration of NAb used (μg/ml) | | NAb | Concentration of NAb used (μg/ml) | |
| --- | --- | --- | --- | --- | --- |
|  | O/Mya/98 | O/Tibet/99 |  | O/Mya/98 | O/Tibet/99 |
| * pOA-1 | 0.005 | 0.005 | pO18-20 | 0.078 | 0.078 |
| pO18-39 | 0.020 | 0.005 | pO18-26 | 0.039 | 0.039 |
| pO18-40 | 0.005 | 0.005 | pO18-16 | 5.000 | 0.156 |
| pOA-7 | 0.005 | 0.010 | B73 | 5.000 | 2.5 |
| pOA-20 | 0.010 | 0.010 | pOA-2 | 0.020 | 0.020 |
| pOA-13 | 0.039 | 0.020 | * pO18-12 | 0.313 | 0.313 |
| pOA-6 | 0.078 | 0.078 | C4 | 0.039 | 0.020 |
| * B66 | 0.156 | 0.156 | F169 | 0.156 | 0.156 |
| * pO18-10 | 1.250 | 0.625 | pO18-2 | 0.010 | 0.010 |
| pO18-17 | 2.500 | 1.250 | pO18-28 | 0.010 | 0.010 |
| B57 | 0.625 | 0.313 | pO18-8 | 0.313 | 0.313 |
| F28 | 0.3125 | 0.3125 | pO18-54 | 1.250 | 1.250 |
| * B82 | 0.078 | 0.020 | * pO18-11 | 0.313 | 0.313 |
| pO18-24 | 1.250 | 0.625 |  |  |  |

Note: Asterisks * indicating the antibodies with the highest affinity to different sites.

**Table S5. Concentrations of 8 strains of neutralizing antibodies cannot be used in cELISA to inhibit the binding of Biotin-NAbs.**

| NAb | Concentration of NAb used(μg/ml) | |  | Inhibition of Biotin-NAb binding (%) | |
| --- | --- | --- | --- | --- | --- |
|  | O/Mya/98 | O/Tibet/99 |  | O/Mya/98 | O/Tibet/99 |
| A19 | 1.250 | 1.250 |  | 0 | -2 |
| B74 | 1.250 | 1.250 |  | 13 | -4 |
| B77 | 5.000 | 5.000 |  | 52 | 52 |
| C5 | 10.000 | 10.000 |  | 2 | 0 |
| E18 | 0.3125 | 0.625 |  | 54 | 51 |
| pO18-52 | 2.500 | 2.500 |  | 49 | 50 |
| pO18-53 | 1.250 | 2.500 |  | 56 | 52 |
| pO18-57 | 10.000 | 10.000 |  | 4 | 4 |

**Table S6. Average antibody valence (log10) detected by site-directed cELISA (six Biotin-NAbs), LPBE and VNT in polyclonal sera from vaccinated cattle.**

| Antigen | Time (dpv) | Serum sources | B66 | pOA-1 | pO18-12 | pO18-11 | B82 | pO18-10 | LPBE | VNT |
| --- | --- | --- | --- | --- | --- | --- | --- | --- | --- | --- |
| O/Mya/98 | 21 | O/SCGH/2016 | 0.79 | 1.53 | 1.47 | 1.69 | 1.55 | 2.10 | 2.62 |  |
|  |  | O/Mya/98 | 1.50 | 1.74 | 1.65 | 1.77 | 1.86 | 2.16 | 2.70 |  |
|  |  | O/Tibet/99 | 1.53 | 1.98 | 1.93 | 1.98 | 1.93 | 2.53 | 2.85 |  |
|  |  | O/XJ/2017 | 1.02 | 1.71 | 1.18 | 1.29 | 1.27 | 2.24 | 2.56 |  |
|  | 49 | O/SCGH/2016 | 1.40 | 1.85 | 1.90 | 2.45 | 2.03 | 2.30 | 3.00 | 2.71 |
|  |  | O/Mya/98 | 1.70 | 2.05 | 2.23 | 2.38 | 2.33 | 2.43 | 3.05 | 2.63 |
|  |  | O/Tibet/99 | 1.78 | 2.15 | 2.49 | 2.49 | 2.54 | 2.75 | 3.14 | 2.65 |
|  |  | O/XJ/2017 | 1.27 | 1.82 | 1.57 | 1.78 | 1.80 | 2.31 | 3.00 | 2.65 |
| O/Tibet/99 | 49 | O/SCGH/2016 | 1.53 | 1.75 | 2.00 | 2.25 | 2.03 | 2.18 | 3.00 | 2.54 |
|  |  | O/Mya/98 | 1.58 | 1.93 | 2.20 | 2.13 | 2.28 | 2.48 | 3.05 | 2.48 |
|  |  | O/Tibet/99 | 1.82 | 2.10 | 2.52 | 2.26 | 2.49 | 2.61 | 3.14 | 2.77 |
|  |  | O/XJ/2017 | 1.36 | 1.80 | 1.87 | 1.73 | 1.82 | 2.38 | 3.00 | 2.8 |

**Table S7. Average antibody valence (log10) detected by site-directed cELISA (six Biotin-NAbs), LPBE and VNT in polyclonal sera from vaccinated sheep.**

| Antigen | Time (dpv) | Serum sources | B66 | pOA-1 | pO18-12 | pO18-11 | B82 | pO18-10 | LPBE | VNT |
| --- | --- | --- | --- | --- | --- | --- | --- | --- | --- | --- |
| O/Mya/98 | 21 | O/SCGH/2016 | 0.10 | 0.85 | 0.48 | 0.63 | 0.50 | 1.53 | 1.78 |  |
|  |  | O/Mya/98 | 0.05 | 0.60 | 0.45 | 0.48 | 0.50 | 1.20 | 1.23 |  |
|  |  | O/Tibet/99 | 0.65 | 1.15 | 0.69 | 0.88 | 0.72 | 1.96 | 2.01 |  |
|  |  | O/XJ/2017 | 0.25 | 1.43 | 0.65 | 0.58 | 0.78 | 2.08 | 2.38 |  |
|  | 49 | O/SCGH/2016 | 0.50 | 1.35 | 1.15 | 1.90 | 1.28 | 1.83 | 2.73 | 2.03 |
|  |  | O/Mya/98 | 0.75 | 1.15 | 1.18 | 1.45 | 1.28 | 1.63 | 2.25 | 1.43 |
|  |  | O/Tibet/99 | 1.62 | 1.87 | 1.59 | 1.98 | 1.82 | 2.54 | 2.84 | 2.30 |
|  |  | O/XJ/2017 | 1.00 | 2.08 | 1.40 | 1.40 | 1.53 | 2.45 | 2.93 | 2.60 |
| O/Tibet/99 | 49 | O/SCGH/2016 | 0.65 | 1.35 | 1.23 | 1.6 | 1.33 | 1.83 | 2.73 | 1.70 |
|  |  | O/Mya/98 | 0.75 | 1.18 | 1.33 | 1.25 | 1.40 | 1.70 | 2.25 | 1.18 |
|  |  | O/Tibet/99 | 1.66 | 1.94 | 1.80 | 2.03 | 1.94 | 2.56 | 2.84 | 2.57 |
|  |  | O/XJ/2017 | 1.28 | 2.13 | 1.50 | 1.58 | 1.53 | 2.58 | 2.93 | 2.84 |

**Table S8. Average antibody valence (log10) detected by site-directed cELISA (six Biotin-NAbs), LPBE and VNT in polyclonal sera from vaccinated pigs.**

| Antigen | Time (dpv) | Serum sources | B66 | pOA-1 | pO18-12 | pO18-11 | B82 | pO18-10 | LPBE | VNT |
| --- | --- | --- | --- | --- | --- | --- | --- | --- | --- | --- |
| O/Mya/98 | 21 | O/SCGH/2016 | 0.00 | 0.39 | 0.42 | 0.9 | 0.60 | 0.9 | 1.83 |  |
|  |  | O/Mya/98 | 0.51 | 0.99 | 0.66 | 1.41 | 0.99 | 1.56 | 1.92 |  |
|  |  | O/Tibet/99 | 1.11 | 1.41 | 0.63 | 1.65 | 1.26 | 2.43 | 2.85 |  |
|  |  | O/XJ/2017 | 0.12 | 0.81 | 0.39 | 0.48 | 0.57 | 1.38 | 1.77 |  |
|  | 49 | O/SCGH/2016 | 0.30 | 1.29 | 0.96 | 2.13 | 1.47 | 1.77 | 2.88 | 1.8 |
|  |  | O/Mya/98 | 1.65 | 2.25 | 1.80 | 2.16 | 2.10 | 2.91 | 2.97 | 2.72 |
|  |  | O/Tibet/99 | 2.16 | 2.43 | 2.64 | 2.94 | 2.79 | 3.12 | 3.42 | 2.82 |
|  |  | O/XJ/2017 | 0.75 | 1.89 | 1.32 | 1.62 | 1.47 | 2.46 | 2.85 | 2.61 |
| O/Tibet/99 | 49 | O/SCGH/2016 | 0.54 | 1.02 | 1.17 | 1.95 | 1.56 | 1.59 | 2.88 | 1.43 |
|  |  | O/Mya/98 | 1.59 | 1.80 | 1.92 | 2.16 | 2.07 | 2.67 | 2.97 | 2.39 |
|  |  | O/Tibet/99 | 2.10 | 2.31 | 2.7 | 2.79 | 2.82 | 3 | 3.42 | 3.02 |
|  |  | O/XJ/2017 | 0.99 | 1.74 | 1.62 | 1.56 | 1.56 | 2.34 | 2.85 | 2.63 |
